# Pathogen effector tactics to suppress plant endomembrane system

**DOI:** 10.3389/fpls.2026.1776548

**Published:** 2026-03-20

**Authors:** Solhee In, Hye-Young Lee, Jongchan Woo, Doil Choi, Eunsook Park

**Affiliations:** 1 Department of Molecular Biology, College of Agricultural, Life Sciences and Natural Resources, University of Wyoming, Laramie, WY, United States; 2 Department of Agriculture, Forestry and Bioresources, Plant Genomics and Breeding Institute, College of Agriculture and Life Sciences, Seoul National University, Seoul, Republic of Korea; 3 Plant Immunity Research Center, College of Agriculture and Life Sciences, Seoul National University, Seoul, Republic of Korea; 4 Department of Horticulture, Gyeongsang National University, Jinju, Republic of Korea; 5 Institute of Agriculture & Life Science, Gyeongsang National University, Jinju, Republic of Korea; 6 Innocorelix, LLC., Laramie, WY, United States

**Keywords:** chloroplast, endoplasmic reticulum, organelle-mediated susceptibility, pathogen effector, plant endomembrane system

## Abstract

Plants have evolved sophisticated defense mechanisms to counter pathogen invasion, including the production of antimicrobial compounds, regulation of defense-related protein expression, and the synthesis of defense hormones across various subcellular organelles. While the significant contribution of organelle functions in plant immunity is increasingly recognized, the specific roles of these organelles in the immune response remain poorly understood. Recent studies have revealed that pathogen effectors from diverse microbes such as fungi, oomycetes, and bacteria localize within various organelles. These effectors target host proteins to manipulate the plant immune system, underscoring the crucial role of organelle functions in plant immunity. This review not only focuses on the localization of effectors within subcellular organelles, excluding the nucleus, but also explores the implications of organelle functions in the plant immune response. Gaining a deeper understanding of how these effectors interact with their targets in specific organelles will pave the way for developing disease-resistant plants.

## Introduction

Plant pathogens usually acquire nutrients and establish a favorable environment within their hosts in order to survive and proliferate. To achieve this, plant pathogens have evolved a wide array of infection strategies that enable them to overcome host physical barriers, establish intimate contact with host cells, and exploit cellular machinery for their proliferation (Pfeilmeier et al., 2016; Rodriguez-Moreno et al., 2018). These strategies often include stomatal hijacking, secretion of cell wall-degrading enzymes to facilitate tissue penetration, formation of specialized feeding structures such as haustoria or extracellular vesicles to access host-derived nutrients, and the deployment of effector proteins that interfere with immune signaling and reprogram host metabolic pathways (Kemen and Jones, 2012; Pfeilmeier et al., 2016; Toruno et al., 2016; Lai et al., 2023). Collectively, these mechanisms allow pathogens not only to invade host tissues and secure essential resources, but also to remodel the host cellular environment, facilitating successful colonization and disease development (Lapin and Van den Ackerveken, 2013; Gorshkov and Tsers, 2022).

However, plants are not acting as a passive target. They are equipped with a multilayered immune system, including pattern-triggered immunity (PTI) and effector-triggered immunity (ETI), which efficiently detect pathogen invasion and activate robust defense responses (Dodds and Rathjen, 2010; Bentham et al., 2020; Jian et al., 2024). Therefore, the outcome of infection is determined by a dynamic arms race that pathogens continuously refine their pathogenicity and virulence strategies to overcome host immunity (Boller and Felix, 2009; Boller and He, 2009; Anderson et al., 2010).

Among the diverse virulence factors employed by pathogens, effector proteins play a central role in establishing host-pathogen interactions (Boller and He, 2009; Hogenhout et al., 2009; Toruno et al., 2016). These molecules are secreted into the host apoplast or extrahaustorial matrix, with some subsequently translocating into host cells to suppress immune signaling pathways and reprogram host metabolism (Lee et al., 2013; Toruno et al., 2016). Many bacterial type III effectors and fungal cytoplasmic effectors directly inhibit pattern recognition receptor (PRR)-mediated signaling, thereby dampening PTI, and some have been shown to suppress ETI (Dodds and Rathjen, 2010; Derevnina et al., 2021). They also target key immune components of downstream signaling cascades including mitogen-activated protein kinase (MAPK) cascades, calcium signaling, reactive oxygen species (ROS) production, and phytohormone signaling (Dou and Zhou, 2012; Meng and Zhang, 2013; Tariqjaveed et al., 2021; Galaud et al., 2025). These activities enable pathogens to efficiently evade host surveillance and establish a permissive environment for their colonization.

Importantly, nutrient uptake is also a fundamental necessity for all classes of plant pathogens, but the strategies differ depending on their lifestyle (Lo Presti et al., 2015). Biotrophic pathogens maintain host plant cells alive and often deploy effectors to subtly reprogram host metabolism, for instance by upregulation of sugar efflux transporters (e.g., SWEET proteins) or modulating amino acid flux through the formation of haustoria (Gupta et al., 2021; Yao et al., 2025). In contrast, necrotrophic pathogens trigger extensive host cell death through toxins, ROS and necrosis-inducing effectors, thereby acquiring nutrients from dead host tissues (Horbach et al., 2011; Mengiste, 2012). Hemibiotrophs combine both strategies, initially behaving as biotrophs before switching to a necrotrophic phase. Through these diverse mechanisms, effectors not only suppress host immunity but also ensure a sustained supply of nutrients, promoting successful pathogen colonization and virulence.

**Figure 1:**
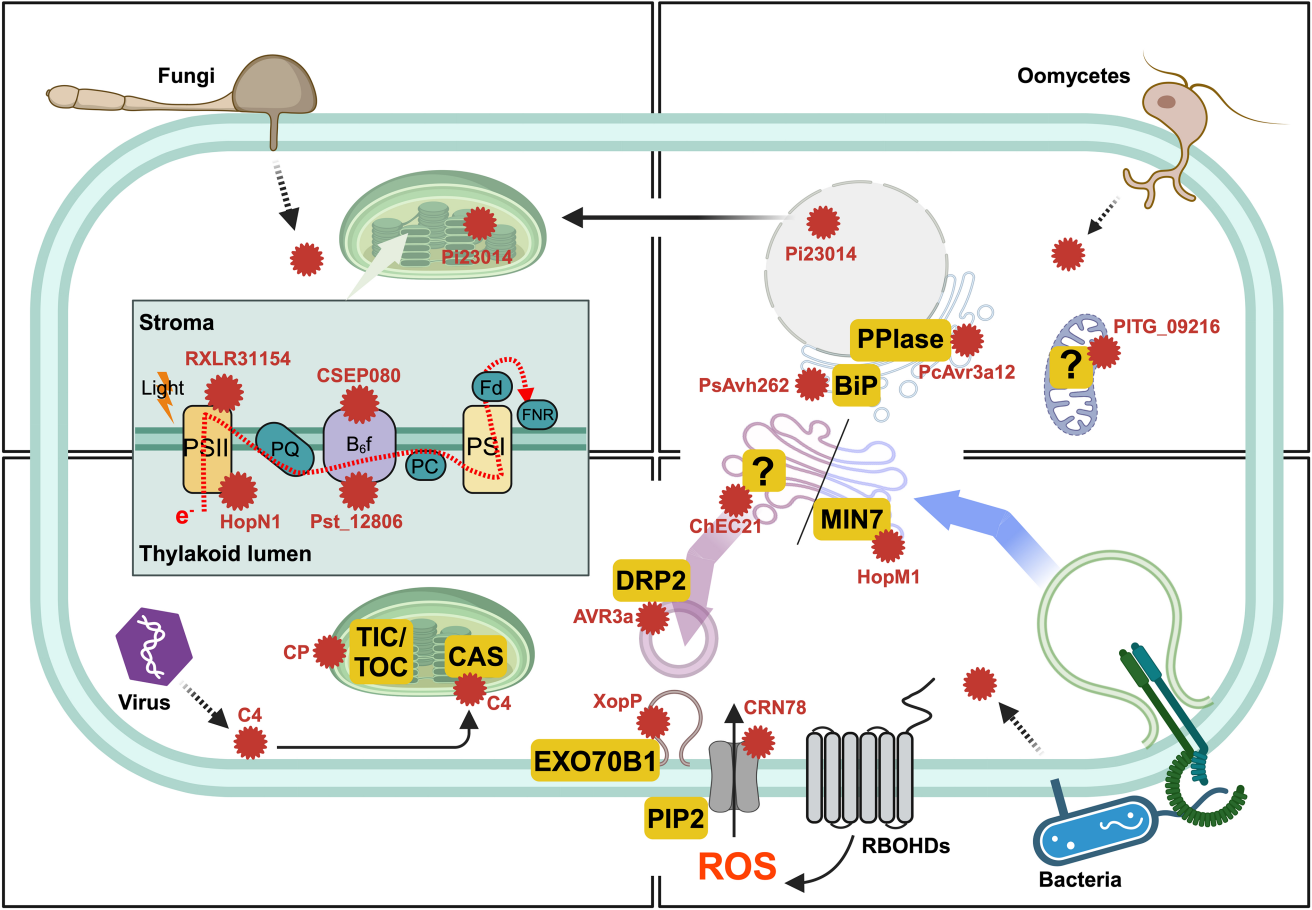
The summary of pathogen effector targets in plant during infection. Various pathogen species secrete the effector proteins to disrupt organelle functions using their own secretion system such as haustoria or type III secretion system.

Beyond immunity suppression and nutrient acquisition, a growing area of research highlights the remarkable ability of effectors to specifically target host organelles. By hijacking host targets across diverse subcellular membrane compartments, pathogen effectors can interfere with central processes such as transcriptional regulation, photosynthesis, ROS production, hormone signaling, and protein secretion, resulting in altered host physiology that create a cellular environment favorable for pathogen colonization. In this review, we focus on subcellular compartment-targeting effectors except the nucleus, summarizing their molecular interactions with host proteins, the cellular functions of the targets disrupted, and how these activities ultimately contribute to pathogenicity. The pivotal roles of nuclear-targeting and membrane trafficking-related effectors in plant immune modulation are not covered in this review, as they have recently been comprehensively described in (Harris et al., 2023; Yuen et al., 2023). In this review, we discuss selective effectors based on two criteria: first, effectors with host localization supported by microscopic evidence, and second, effectors that promote pathogen virulence or suppress hypersensitive response (HR) cell death in model plants. Guided by these criteria, we compiled key information such as host targets and identification methods (**Tables 1**, **2**) to illustrate the functional relevance of organelle-localized effectors (**Figure 1**).

**Table 1:** ER, Golgi and PM targeting effectors and its host targets. Further details are provided in the **Supplemental Table 1**.

Effector name	Pathogen spp	Effector localization	Host target	Effector function	Host target identification	Interaction validation	Reference
Pc12	*Phytophthora capsici*	ER, Golgi	Rab13	interfere with Rab13-2 trafficking	IP-LC-MS/MS	Y2H, Co-IP	Kim et al., 2024
PhRXLR-C13	*Plasmopara halstedii*	ER	nd	nd	nd	nd	Breeze et al., 2023
PhRXLR-C21							
PhRXLR-C22							
PITG_04367	*Phytophthora infestans*	ER					
PITG_09223							
PITG_13044							
PITG_13048							
PITG_15297							
PITG_15318							
PITG_13045		ER, Golgi					
HaRXLL492	*Hyaloperonospora arabidopsidis*	ER					
HaRXLL493d							
HaRXLL495c							
RXLR242	*Phytophthora capsici*	ER, nucleus	RabE1-7	interfere the defense-related protein secretion pathway	IP-LC-MS/MS	BiFC, Co-IP, Pull down	Li et al., 2022
PbPE	*Plasmodiophora brassicae*	ER, nucleus	nd	suppression of INF1/NPP1-mediated cell death	nd	nd	Hossain et al., 2021
PbPE2							
PbPE10							
PbPE14							
PbPE17		ER, Golgi					
PbPE1		ER, Golgi, nucleus					
PbPE3		cis-faced Golgi					
MoCDIP4	*Magnaporthe oryzae*	ER	DjA9	promotes the DRP1E accumulation to promote mitochondra fission	Y2H screening	Y2H, Pull down BiFC, LCI	Xu et al., 2020
RipD	*Ralstonia solanacearum*	ER	nd	suppression of ROS burst, defense related gene expression	nd	nd	Jeon et al., 2020
RipN	*Ralstonia solanacearum*	ER, nucleus	nd	converts NADH/NAD+ ratio, promote bacterial growth, suppresses PTI	nd	nd	Sun et al., 2019
PcAvr3a12	*Phytophthora capsici*	ER network, nucleus perinuclear ER, cytoplasm	FKBP15-2	suppression of PPIase activity	Y2H screening	BiFC, Y2H, Co-IP	Fan et al., 2018
PvRXLR80	*Plasmopara viticola*		nd	nd	nd	nd	Liu et al., 2018
PsAvr262	*Phythophthora sojae*	ER, haustoria, nucleus	NbBiP5, GmBiP1/4	stabilization of BiP, suppressing ER stress mediated cell death	IP-LC-MS/MS	BiFC, Co-IP, pull down	Jing et al., 2016
PITG_15235	*Phytophthora infestans*	Golgi	nd	nd	nd	nd	Breeze et al., 2023
PITG_23046							
PITG_23202							
PITG_14797							
ChEC21	*Collectotrihum higginsianum*	Golgi	nd	nd	nd	nd	Robin et al., 2018
HopM1	*Pseudomonas syringae* DC3000	TGN/EE	MIN7	MIN7 degradation promotion	Y2H screening	Y2H, Pull down	Nomura et al., 2011
AVRblb2	*Phytophthora infestans*	Plasma membrane	cysteine protease C14, CaM, CML	prevents the C14 apoplast secretion, inhibiton of CaM-CML-CNGC dissociation	IP-LC-MS/MS Y2H screening	Co-IP, Y2H, BiFC, Pull down	Bozkurt et al., 2011; Lee et al., 2024
CRISIS2	*Phytophthora capsici*	Plasma membrane	Plasma membrane H^+^-ATPase	inhibition of PMA activity	effector screening	Co-IP, Y2H	Seo et al., 2023
XopP	*Xanthomonas campestris*	Plasma membrane	Exo70B	suppression or inhibition of PTI-related protein transport	Y2H screening	BiFC, Y2H, Co-IP, SEC	Michalopoulou et al., 2022
AvrE	*Pseudomonas syringae* DC3000	Plasma membrane, PM-associated vesicle	Type one protein phosphatase	ABA signaling manipulation	Pull down with MS	LCA, Co-IP, Pull down	Hu et al., 2022
CRN78	*Phytophthora sojae*	Plasma membrane	PIP2;2	promote the PIP2;2 degradation	IP-LC-MS/MS	BiFC, Co-IP LCA	Ai et al., 2021
PsAvh240	*Phytophthora sojae*	Plasma membrane	GmAP1	inhibits GmAP1 apoplast secreation	IP-LC-MS/MS	Co-IP	Guo et al., 2019
PvRXLR154	*Plasmopara viticola*	Plasma membrane	nd	nd	nd	nd	Liu et al., 2018
HopAF1	*Pseudomonas syringae* DC3000	Plasma membrane	MTN1, MTN2	disruption of PAMP-induced ethylene biosynthesis	Y2H screening	BiFC, Co-IP	Washington et al., 2016
HopF2	*Pseudomonas syringae* DC3000	Plasma membrane	BAK1	PTI signaling suppression		Co-IP, BiFC, Pull down	Zhou et al., 2014
XopJ	*Xanthomonas campestris* pv.*vesicatoria*	Plasma membrane	Proteasome 19S subunit RPT6	decrease of proteasome activity	Y2H screening	BiFC,Co-IP, Y2H, Pull down	Ustun et al., 2013
AvrPto	*Pseudomonas syringae* DC3000	Plasma membrane	BAK1	inhibition of FLS2-BAK1 complex formation		Co-IP, Pull down yeast split Ub assay	Shan et al., 2008

IP-LC-MS/MS, Immunoprecipitation-liquid chromatography-tandem mass spectrometry; Y2H, Yeast-two hybrid; Co-IP, Co-immunoprecipitation; BiFC, Bimolecular fluorescence complementation; SEC, Size exclusion chromatograph; LCA, Luciferase complementation assay; Ub, Ubiquitin.

**Table 2:** Chloroplast, mitochondria and peroxisome targeting effectors and its host targets. Additional information is available in the **Supplemental Table 1**.

Effector name	Pathogen spp	Effector localization	Host target	Effector function	Host target identification	Interaction validation	Reference
cC4	*Tomato golden mosaic virus*	Chloroplast, nucleus, cytosol	Toc64-III, PUB4	promotes chloroplast degradation	IP-MS	BiFC, Y2H	Mei et al., 2024
Pi23014	*Phytophthora infestans*	Chloroplast, nucleus	RBP3a	nd	IP-LC-MS/MS	BiFC, Y2H, Co-IP	Li et al., 2024
SRP1	*Candidatus Phytoplasma asteri*	Chloroplast	Glutamine synthase	suppression of glutamine synthase oligomer formation	Y2H screening	BiFC, Y2H, Co-IP Pull down	Zhang X et al., 2024
CP (coat protein)	*Melon necrotic spot virus*	Chloroplast, mitochondria	TOC159A/B, TOM20-1/2	nd	nd	BiFC	Saiz-Bonilla et al., 2023
CSEP080	*Erysiphe necator*	Chloroplast, plasma membrane	VviB6f in chloroplast, VviPE in plasma membrane	affects photosynthesis, inhibits INF1 cell death	Y2H screening	BiFC, Y2H, Co-IP	Mu et al., 2023
PmEC04572	*Phyllachora maydis*	Chloroplast	nd	nd	nd	nd	Helm et al., 2022
RsCRP1	*Rhizoctonia solani*	Chloroplast, mitochondria	nd	nd	nd	nd	Tzelepis et al., 2021
RXLR31154	*Plasmopara viticola*	Chloroplast, cytosol, nucleus	OEE2 or PsbP	PsbP stabilization	Y2H screening	BiFC, Y2H, Co-IP	Liu et al., 2021
C4	*Tomato yellow leaf curl virus*	Chloroplast, plasma membrane	CAS	suppress the SA biosynthesis	AP-MS	BiFC, Co-IP	Medina-Puche et al., 2020
EqCSEP01276	*Erysiphe quercicola*	(sort of) Chloroplast, cytosol	NCED5	suppresses ROS accumulation, inhibits ABA biosynthesis	Pull down, LC-MS/MS	BiFC, Y2H, Co-IP	Li et al., 2020
PstCTE1	*Puccinia striiformis f.* sp. *tritici*	nd	nd	nd	nd	nd	Andac et al., 2020
RipAD	*Ralstonia solanacearum*	Chloroplast	nd	suppression of ROS burst, defense related gene expression	nd	nd	Jeon et al., 2020
SsITL	*Sclerotinia sclerotiorum*	Chloroplast	CAS	CAS dependent SA-signaling pathway	IP-LC-MS/MS	Y2H, Co-IP, Pull down	Tang et al., 2020
Pst_12806	*Puccinia striiformis f.* sp. *tritici*	Chloroplast	TaISP	suppresses BAX-induced cell death, callose deposition	Y2H screening	BiFC, Y2H, Co-IP	Xu et al., 2019
PvRXLR61	*Plasmopara viticola*	Chloroplast, nucleus	nd	nd	nd	nd	Liu et al., 2018
PvRXLR86		Chloroplast					
PvRXLR161		Chloroplast, nucleus					
PvRXLR54		Chloroplast, nucleus, mitochondria					
HopR1	*Pseudomonas syringae* DC3000	Chloroplast, cytosol	nd	suppressing the HopQ1 cell death	nd	nd	Zembek et al., 2018
	*Pseudomonas syringae* pv. *phaseolicola*						
RipAL	*Ralstonia solanacearum*	Chloroplast	nd	modulates JA signaling	nd	nd	Nakano and Mukaihara, 2018
HopN1	*Pseudomonas syringae* pv. *actinidiae*	Chloroplast	nd	nd	nd	nd	Choi et al., 2017
	*Pseudomonas syringae* DC3000		PsbQ	inhibits PSII activity	Pull down, LC-MS/MS	Pull down	Rodriguez-Herva et al., 2012
HopK1	*Pseudomonas syringae* DC3000	Chloroplast	nd	suppresses PTI	nd	nd	Li et al., 2014
Mlp107772	*Melampsora larici-populina*	Chloroplast, mitochondria	nd	nd	nd	nd	Petre et al., 2016
PITG_09216	*Phytophthora infestans*	Mitochondria	nd	nd	nd	nd	Breeze et al., 2023
PITG_09218							
PITG_22884							
AvrPita	*Magnaporthe oryzae*	Mitochondria	COX11	suppression of ROS	Y2H screening	Y2H, Pull-down, LCA	Han et al., 2021
HopG1	*Pseudomonas syringae* DC3000	Mitochondria	nd	suppresses the mitochondrial respiration and ROS accumulation	nd	nd	Block et al., 2010
PpE18	*Phytophthora parasitica*	Peroxisome, ER	NbAPX3-1, NbANKr2	inhibits NbAPX3-1 ROS scavinging activity	IP-LC-MS/MS	Co-IP, Pull-down	Cao et al., 2024
ChEC51a	*Collectotrihum higginsianum*	Peroxisome	nd	nd	nd	nd	Robin et al., 2018
ChEC89							
ChEC96							
γb	*Barley stripe mosaic virus*	Peroxisome	GOX	inhibits ROS accumulation, GOX activity	IP-LC-MS/MS	Co-IP, BiFC, Pull-down, gel filtration assay	Yang et al., 2018
RipAK	*Ralstonia solanacearum*	Peroxisome	AtCAT1~3	inhibits CAT activity	Pull down	Y2H, BiFC	Sun et al., 2017
P8	*Rice dwarf phytoreovirus*	Peroxisome	GOX	nd	Y2H screening	Y2H, Co-IP	Zhou et al., 2007

IP-LC-MS/MS, Immunoprecipitation-liquid chromatography-tandem mass spectrometry; Y2H, Yeast-two hybrid; Co-IP, Co-immunoprecipitation; BiFC, Bimolecular fluorescence complementation.

## Plant endomembrane system is the most attractive target of the effectors

### Endoplasmic reticulum

The endoplasmic reticulum (ER) is a central organelle responsible for lipid biosynthesis, protein synthesis, folding, removal of misfolded proteins, and protein secretion. Under stress conditions, the demand for plasma membrane receptor proteins and secreted proteins markedly increases and can exceed the ER’s folding and processing capacity, leading to ER stress (Angelos et al., 2017). This imbalance triggers the unfolded protein response (UPR), leading to the activation of the ER-quality control system by upregulating molecular chaperones and folding enzymes, while simultaneously inducing ER-associated degradation machinery to remove misfolded proteins (Eichmann and Schafer, 2012; Howell, 2013).

However, when environmental or developmental cues impose extreme stress that exceeds the buffering capacity of the ER quality-control system, programmed cell death (PCD) can be triggered (Howell, 2013). Pathogen effectors target the host ER stress pathway and manipulate it to their advantage during infection (**Table 1**). Cell death can either harm or benefit pathogens, depending on its timing and the pathogen’s ability to exploit dying tissues (Dickman and Fluhr, 2013; Jing and Wang, 2020). The intracellular obligate biotrophic plant pathogen, *Plasmodiophora brassicae*, infects canola by secreting putative effectors (Hossain et al., 2021). Although 12 of the 15 candidates lack an ER retention signal, they localize predominantly to the ER when expressed in *Nicotiana benthamiana*, suggesting their roles in manipulating plant cell secretion and vesicle trafficking (Hossain et al., 2021). Among them, several effectors inhibit PCD induced by *Phytophthora infestans* INF1- or NPP1 (Necrosis-inducing Phytophthora protein 1), but how they suppress cell death and manipulate ER stress remains unknown.

Two of the conserved effectors across different *Phytophthora capsici* strains, RXLR242 and Pc12 targets to the ER-Golgi interface (Li et al., 2022; Kim et al., 2024, respectively). RXLR242 is localized at ER and in nucleus, but NLS-GFP-RXLR242 loses its virulence, indicating that ER membrane localization is a prerequisite for RXLR242-associated virulence. Comparative mass spectrometry analysis has revealed that RXLR242 and Pc12 physically associate with a group of RAB proteins that belong to the small GTPase family in *N. benthamiana* and disrupts their function for infection (Li et al., 2022; Kim et al., 2024). RXLR242 inhibits the association between RABA4–3 and its binding protein related to vesicle, disrupting the trafficking of the membrane receptor FLAGELLIN-SENSING 2 (FLS2). Therefore, it appears to be an effective virulence strategy for pathogen effectors to modulate host ER function. On the other hand, Pc12 interacts with RAB13 proteins and stabilizes their interaction with GDI, potentially inhibiting the dynamics of RabGTPase activity, preventing ER-Golgi trafficking broadly (Kim et al., 2024). The bottleneck of membrane trafficking at the ER-Golgi interface raises the ER stress, potentially induces the necrotic cell death (Kim et al., 2024).

During ER stress, several membrane-associated transcription factors are translocated from the ER to the nucleus for transcriptional reprogramming involved in ER stress responses, including programmed cell death (Kim et al., 2007; Yang et al., 2014). Since global transcriptional reprogramming affects the secretion of defense-related proteins and the induction of systemic acquired resistance (SAR) (Moreno et al., 2012; Nagashima et al., 2014), this process is a favorable target, which effectors subvert to alter protein localization, facilitating a cellular environment for disease development. Some Tail-anchored RXLR effectors of oomycete *Hyaloperonospora arabidopsidis* and *Plasmopara halstedii* predominantly localize to the ER lumen of which effectors are capable of interacting with membrane-localized NAC transcription factors (Breeze et al., 2023). In addition, multiple RXLR effectors from diverse pathogens can be associated with a subset of NACs implicated in UPR and PCD signaling pathways, suggesting that oomycetes employ effectors to regulate the transcription of genes associated with the UPR and PCD.

Compared with other organelles, relatively few effectors have been characterized that specifically target the ER. However, given the critical role of the ER as a signal transducer of environmental stress, pathways such as UPR and ER quality control system appear to be major targets of pathogen manipulation for host-microbe compatibility. Therefore, identifying effector targets that modulate ER stress signaling or ER stress-related PCD will provide valuable insights into ER-mediated susceptibility and their contribution to plant-pathogen interactions.

### Golgi

From the ER, some proteins and lipids are subsequently delivered to the Golgi apparatus, which functions as the next central hub in the endomembrane trafficking system. In plants, the Golgi apparatus is responsible not only for synthesizing cell wall polysaccharides and plasma membrane glycolipids, but also for glycosylating proteins destined for the cell wall, plasma membrane, or storage vacuoles (Neumann et al., 2003). The Golgi serves as the principal sorting and trafficking station, targeting proteins to their proper cellular destinations. Newly synthesized and endocytosed cargoes are sorted at the trans-Golgi network/early endosome (TGN/EE), which serves as the key hub for directing them to the plasma membrane or vacuole (LaMontagne and Heese, 2017). This functional connection from the ER to the Golgi underscores the coordinated regulation of protein and lipid processing in support of plant growth, development, and stress responses. Because the Golgi apparatus plays pivotal roles in vesicle trafficking for normal plant growth and defense, such as antimicrobial protein transportation, recognition of effector protein by Golgi-localized receptor (Uemura et al., 2012; Bhandari et al., 2023; Wang et al., 2025), it appears to be a major target organelle for pathogen effectors (**Table 1**). Nevertheless, compared with other organelles, relatively few effectors targeting the Golgi have been identified to date. Among the Golgi-targeted effectors identified, the *P. syringae* DC3000 effector HopM1 has been the most extensively characterized. HopM1, one of the most highly conserved type III effectors of *P. syringae*, has been shown to localize to the TGN/EE compartment, as demonstrated by co-localization with a TGN/EE marker, VHA-a1 (Nomura et al., 2011). At the TGN/EE, HopM1 binds multiple MIN proteins, key regulators of vesicle trafficking. Among them, the well-characterized MIN7 is an ARF guanine-nucleotide exchange factor that activates ARF family GTPases by catalyzing GDP-GTP exchange, facilitating PTI, ETI, and SA-mediated immunity in Arabidopsis (Nomura et al., 2006, 2011). In *Pseudomonas*-infected cells, HopM1 degrades MIN7 via the 26S proteasome to disrupt vesicle trafficking, weakening host immunity (Nomura et al., 2006).

Although experimental evidence from co-localization studies demonstrated Golgi targeting of several *P. infestans* and *Colletotrichum higginsianum* effectors, the detailed mechanisms by which they contribute to pathogen virulence remain unclear (Robin et al., 2018; Breeze et al., 2023).

### Plasma membrane

Plants have established sophisticated surveillance mechanisms to sense danger signals at the cell surface and promptly activate immunity. The plasma membrane of host plant cells functions as the frontline in both plant defense and pathogen attack, harboring a diverse repertoire of PRRs and co-receptors that detect pathogen-associated molecular patterns or damage-associated molecular patterns to activate basal defense responses. This strategic positioning of PRRs at the cell surface ensures rapid immune perception, enabling the initiation of downstream signaling cascades that form the first layer of plant immunity. Thus, from the pathogen’s perspective, deployment of effector proteins that suppress these early-stage basal defenses at the plasma membrane might be an effective and efficient strategy for successful colonization. Indeed, several effectors localize to the plasma membrane, where they interfere with either PRRs or their co-receptors to attenuate defense responses (**Table 1**; Wu et al., 2011; Zhou et al., 2014; Tang et al., 2017). These findings reinforce the view that the plasma membrane constitutes a critical target organelle for pathogen effectors. One example is AvrPto. AvrPto is a type III effector from *P. syringae* pv. *tomato* that localizes to the plasma membrane and targets the PRRs such as FLS2 and EFR, as well as their co-receptor BAK1 (Shan et al., 2008; Xiang et al., 2008). It inhibits autophosphorylation of FLS2 and EFR, suppressing PTI (Xiang et al., 2008).

In addition to plasma membrane-associated receptors, the plasma membrane harbors various other components such as secondary transporters involved in immune responses, which also serve as major targets of pathogen effectors. The *P. infestans* effector AVRblb2 targets cyclic nucleotide-gated channels (CNGCs) in the plasma membrane, which regulate Ca²^+^ influx and plays a pivotal role in downstream immune signaling and cellular responses. By interfering with the CNGC function, pathogen effectors disrupt intracellular calcium signaling, suppressing ROS production and attenuating immune responses (Lee et al., 2024). Plasma membrane H^+^-ATPases (PMAs) are primary pumps that establish an electrochemical gradient across the PM and are essential for generation of cell membrane potential. *P. capsici* RXLR effector CRISIS2 interacts with and irreversibly inhibits PMAs in host plants, thereby inducing host cell death (Seo et al., 2023). Since the gene-silencing of *CRISIS2* resulted in smaller necrotic lesions and reduced pathogen biomass, CRISIS2-induced cell death might contribute to the virulence of *P. capsici* for supporting the hemibiotrophic lifestyle of the pathogen (Seo et al., 2023). CRN78, a Crinkler (CRN) family effector from the oomycete *Phytophthora sojae*, is also localized to the plasma membrane (Ai et al., 2021). CRN78 interacts with PIP2-family aquaporin proteins and promotes phosphorylation of PIP2 using its kinase domain, resulting in its degradation via 26S-dependent pathway. Taken together, effectors targeting immune regulators at the plasma membrane appear to be an efficient strategy for evading host immune responses at the early stage of pathogenesis.

Apart from effectors that localize to the plasma membrane through interactions with plasma membrane-associated transporters, several effectors directly anchor to the plasma membrane to modulate membrane integrity and water/nutrient balance, contributing to pathogen virulence. HopAF1 from *P. syringae* targets the plasma membrane via an acylation signal sequence at G2 and C4 (Washington et al., 2016). HopAF1 is associated with the plasma methylthioadenosine nucleosidase proteins MTN1 and MTN2 to dampen ethylene production during bacterial infection. A highly conserved effector family in many phytopathogenic bacteria, AvrE can form water- and ion-permeable channels in the plant plasma membrane to create a hydrated and nutrient-rich extracellular space required for disease establishment (Xin et al., 2015). Although AvrE has been reported to induce water-soaking by manipulating the ABA signaling pathway (Hu et al., 2022), this evidence also suggests a more direct role for AvrE effectors in promoting apoplastic water-soaking. A broader picture emerges in which phytopathogens deploy multiple effectors targeting plasma membrane-resident regulatory constituents that disrupt cellular homeostasis for successful colonization in host plants.

## Effectors target critical biological processes for plant living in other organelles

### Chloroplast

Chloroplasts originated through an ancient endosymbiotic event, in which a photosynthetic cyanobacterium was engulfed by a proto-eukaryote and subsequently integrated as an intact organelle (Slesak and Slesak, 2024). This evolutionary event enabled plants to perform oxygenic photosynthesis, fundamentally shaping the biosphere. During photosynthesis, chloroplasts capture light energy in the thylakoid membrane and convert it into chemical energy through the coordinated activity of photosystems and the electron transport chain (Foyer et al., 2012).

Chlorophyll, a green pigment embedded in the thylakoid membrane, traps red and blue light during photosynthesis while reflecting green wavelengths that confer the characteristic green color of leaves. This green coloration tends to evoke a sense of freshness in humans, whereas many herbivorous insects, such as the Dalbulus leafhopper, are more strongly attracted to yellowing plant leaves (Todd et al., 1990). The pathogens transmitted by piercing–sucking insect vectors frequently exploit this preference by inducing leaf yellowing symptoms in their host plants, thereby facilitating their own transmission. Rice orange leaf phytoplasma (ROLP), a fastidious phloem-limited pathogen of rice that colonizes the phloem sieve elements of the vasculature, provides a well-characterized example of this infection strategy. ROLP secretes an effector, secreted ROLP protein 1 (SRP1), which is delivered from infected sieve elements to adjacent cells and targeted to chloroplasts (Zhang X et al., 2024). SRP1 directly associates with rice glutamine synthetase (OsGS2) to diminish its enzymatic activity. This inhibition compromises the production of key precursors in the chlorophyll biosynthetic pathway such as glutamate, glutamine, and 5-aminolevulinic acid, resulting in reduced chlorophyll accumulation and severe leaf chlorosis. Pathogen-induced chlorosis likely enhances the visual cues that attract vector insects, thereby increasing pathogen virulence and transmission efficiency.

Photosystem II (PSII) generates dioxygen by catalyzing the light-driven splitting of water, releasing electrons and protons (Ago et al., 2016). The oxygen-evolving complex of PSII includes the PsbQ subunit, which stabilizes the extrinsic proteins and optimizes water oxidation, ensuring efficient electron flow to downstream components (Ifuku et al., 2005). Because photosynthesis is vital for plant survival, it frequently becomes a primary target of pathogen infection strategies aimed at modulating host physiology (**Table 2**). The bacterial effector HopN1 from the hemibiotrophic pathogen, *Pseudomonas syringae* specifically targets the chloroplast thylakoid and promotes the degradation of the PsbQ protein, destabilizing the PSII complex. This diminishes the photolysis of water and consequently reduces both oxygen production and electron transport (Rodriguez-Herva et al., 2012). Similarly, the stripe rust fungus (*Puccinia striiformis*) effector Pst_12806 localizes to chloroplasts and interacts with the C-terminal Rieske domain of the wheat TaISP protein, a subunit of Cyt b6/f that connects PSII and PSI in the photosystem, resulting in impaired electron transport and photosynthetic efficiency (Xu et al., 2019).

During photosynthesis, the photosynthetic electron transport chain (PETC) in chloroplasts generates ROS including singlet oxygen (^1^O^2^), superoxide (O_2_^-^), and hydrogen peroxide (H_2_O_2_) as byproducts (Triantaphylides and Havaux, 2009; Li and Kim, 2022). When plants absorb more light energy than can be used for carbon fixation, excess excitation energy leaks to oxygen, increasing ROS production. To prevent this overproduction, plants activate regulatory mechanisms such as photorespiration, cyclic electron flow around PSI or PSII, and the downregulation of PSII efficiency by the xanthophyll cycle and proton gradient (Asada, 2006). These relaxation systems dissipate excess energy, limit ROS formation, and help maintain photosynthetic balance, which is essential because both insufficient control and excessive accumulation of ROS threaten cellular survival. Therefore, the regulatory processes must be precisely coordinated. In other words, chloroplast-derived ROS release associated with ETI and the subsequent hypersensitive response, becomes a powerful mechanism of plants (Su et al., 2018; Cai et al., 2025; Roussin-Leveilee et al., 2025). Thus, pathogen effectors interfere with these regulatory mechanisms to promote pathogen colonization. The powdery mildew (*Erysiphe necator*) effector protein, CSEP080, interacts with grapevine (*Vitis vinifera* L.) chloroplast protein b6f, affecting the photosynthetic electron transport chain between PSII and PSI (Mu et al., 2023). Importantly, Vvib6f was shown to increase photosynthesis capacity while simultaneously reducing hydrogen peroxide accumulation and disease resistance in grapevine, indicating that photosynthetic modulation is directly linked to the suppression of immune responses. By promoting carbon assimilation while attenuating ROS-based immune signaling, effectors such as CSEP080 increase host susceptibility and ultimately compromise plant fitness despite transient increases in photosynthetic activity. Similarly, an RXLR type effector of *Plasmopara viticola* targets chloroplast function by associating with the chloroplast-localized VpPsbP protein, a subunit of the oxygen-evolving enhancer proteins *in planta* (Liu et al., 2021). PsbP resides in the thylakoid lumen, where it modulates the water-splitting reaction and is essential for the assembly and stability of PSII. Notably, PsbP protein levels increase under abiotic conditions such as heat stress in sweet potato and during *P. viticola* infection (Milli et al., 2012). During infection, RXLR31154 may stabilize VpPsbP to suppress H_2_O_2_ production while simultaneously activating the ¹O_2_ signaling pathway for pathogen proliferation (Liu et al., 2021). Through examples of effectors that specifically target chloroplasts, particularly photosystems, it has become evident that pathogens have evolved efficient strategies to promote their colonization by enhancing photosynthetic performance while suppressing the accumulation of ROS as a critical defense signal.

Another reason why chloroplasts are regarded as a pivotal organelle in plant-pathogen interactions is their central role in the biosynthesis of salicylic acid (SA), a phytohormone critical for defense. In many plants, SA is primarily synthesized from chorismate through the chloroplast-localized isochorismate pathway, with isochorismate synthase 1 (ICS1). This pathway provides the bulk of pathogen-induced SA, which is essential for the activation of systemic acquired resistance and the expression of pathogenesis-related genes (Wildermuth et al., 2001; Rekhter et al., 2019). Chloroplasts therefore not only function as sites of photosynthesis but also act as metabolic hubs for generating hormonal defense signals. Pathogen effectors that perturb chloroplast function indirectly interfere with hormone signaling, further weakening plant immunity and promoting susceptibility. Indeed, several pathogen effectors have been reported to localize to chloroplasts and reduce SA levels, inhibiting host defense responses (Jelenska et al., 2007; Nakano and Mukaihara, 2018; Medina-Puche et al., 2020; Tang et al., 2020). SsITL, a secretory protein of the necrotrophic fungus *Sclerotinia sclerotiorum*, interacts with a chloroplast-localized calcium-sensing receptor (CAS) (Tang et al., 2020). CAS, a thylakoid membrane protein, is required for SA biosynthesis, PTI and ETI-induced expression of defense genes, calcium-dependent retrograde signaling, callose deposition, and hypersensitive response (Nomura et al., 2012). SsITL suppresses host immunity by associating with CAS to inhibit its function as a positive regulator in plant immunity at the early stage of *S. sclerotiorum* infection (Tang et al., 2020). Tomato yellow leaf curl virus (TYLCV) protein C4 also associates with CAS to inhibit CAS-dependent defense responses rather than SA perception (Medina-Puche et al., 2020). C4 changes the localization from the plasma membrane to chloroplasts and manipulates the CAS-mediated defense responses such as retrograde signaling during PTI, cytosolic Ca^2+^ burst, and SA biosynthesis (Medina-Puche et al., 2020). As described, CAS in the SA signaling pathway is a common effector target across diverse pathogens, indicating that disruption of the SA biosynthetic cascade provides an advantage for pathogen colonization.

In addition to SA, abscisic acid (ABA) exerts both positive and negative effects on plant immunity depending on the type of pathogen. Several pathogen effectors have evolved to target and modulate components of the ABA signaling cascades for their proliferation in host plants. The ABA biosynthetic process begins in chloroplasts, where 9-cis-epoxycarotenoids are produced as key intermediates (Chen et al., 2020). These compounds are cleaved by 9-cis-epoxycarotenoid dioxygenase (NCED) within the plastid to generate xanthoxin (Qin and Zeevaart, 1999). NCED of rubber trees (*Hevea brasiliensis*) is a target of biotrophic fungus effector EqCSEP01276 from *Erysiphe quercicola* (Li et al., 2020). Because ABA contributes to the plant defense against *E. quercicola* in *H. brasiliensis*, EqCSEP01276 interacts with HbNCED5, a chloroplast-localized protein to disrupt its role in ABA biosynthesis, promoting successful pathogen compatibility (Li et al., 2020). These findings suggest that viral and fungal effectors have evolved to suppress immune response linked to hormone biosynthesis and signaling in the chloroplast.

### Mitochondria and peroxisome

Despite their central roles in cellular metabolism and stress responses, few effectors specifically target mitochondria or peroxisomes (**Table 2**). Mitochondria are key organelle for cellular respiration performed by several multiprotein complexes in the inner mitochondrial membrane, including complex I, II, III and IV, as well as NAD(P)H dehydrogenases. Electron transport via complexes I III and IV is coupled to proton transport and the generation of an electrochemical gradient which is used to drive ATP synthase to produce ATP. In the mitochondrial oxidative phosphorylation process, cytochrome c oxidase (COX) functions as the terminal complex of the electron transport chain, coupling the oxidation of reduced cytochrome c from complex III with the transfer of electrons to the final electron acceptor, oxygen (O_2_) (Ghifari et al., 2023). Among the complex proteins, the COX complex assembly chaperone, COX11 has a role in controlling the timing of the oxidative burst by ROS scavenging (Luo et al., 2013). Necrotrophic pathogen *Rhizoctonia solani* delivers CtaG/COX11, a truncated form of COX11, to increase host susceptibility of *R. solani* in *N. benthamiana* (Zhang D et al., 2024). RsIA_CtaG/Cox11 targets host mitochondria and associates with the rice host (*Oryza sativa*) protein, CoxVIIa. RsIA_CtaG/Cox11 triggers OsCoxVIIa-mediated cell death with increased susceptibility of *N. benthamiana* to *R. solani*, suggesting that RsIA_CtaG/Cox11 may contribute to necrotrophic cell death induction during pathogenesis via regulation of mitochondrial ROS metabolism. Another effector that targets COX is AvrPita from *Magnaporthe oryzae* (Han et al., 2021). AvrPita targets the host mitochondria and also interacts with COX11 in rice. Overexpressing AvrPita or OsCOX11 increased COX activity and suppressed ROS accumulation triggered by the fungal PAMP chitin, indicating that *M. oryzae* secretes AvrPita to the host, where it enhances COX activity and reduces ROS accumulation (Han et al., 2021). Therefore, ROS metabolism in host mitochondria has emerged as a major target exploited by pathogens, as manipulating this process enables them to modulate host ROS levels and facilitate disease development.

Peroxisomes are also key organelles that play an important role in plant ROS metabolism. *Ralstonia solanacearum* effector, RipAK localizes to Arabidopsis peroxisomes and directly interacts with catalases (CATs), which function as ROS scavengers by degrading H_2_O_2_ (Sun et al., 2017). Localization of RipAK to peroxisomes is dependent on its association with CATs and heterologous expression of *RipAK* inhibits CAT activity, demonstrating that *Ralstonia* suppresses the ROS burst by inhibiting host CATs through RipAK during early infection.

Similarly, virus utilizes viral effector proteins to target peroxisomal ROS system. The barley stripe mosaic virus (BSMV) γb protein interacts with the host glycolate oxidase (GOX) which is a flavin mononucleotide-containing enzyme in peroxisomes. It catalyzes oxidation of glycolate or its derivatives and generates glyoxylate and H_2_O_2_ during photorespiration (Yang et al., 2018). This study shows that the γb protein inhibits GOX enzymatic activity to suppress peroxisomal H_2_O_2_ production during BSMV infection (Yang et al., 2018). Moreover, the P8 protein of Rice dwarf phytoreovirus also targets peroxisomes of rice and interacts with rice GOX (Zhou et al., 2007). These findings suggest that pathogens also exploit host peroxisomes or mitochondria, particularly their ROS-producing systems, to promote proliferation in host plants.

## Perspective

Effector biology has revealed that pathogens exploit a wide spectrum of host proteins to suppress immunity and modulate cellular processes in their favor. Many of these effectors converge on host proteins that function as susceptibility (S) factors. Identification of effector targets as candidate S-genes provides an opportunity to uncover vulnerabilities in crops that can be modified to develop durable resistance. However, a successful strategy requires in-depth mechanistic insights into how effectors manipulate target functions in the host without compromising plant fitness. Notably, the host proteins targeted by effectors often fulfill essential roles in normal physiological processes, suggesting that they represent particularly useful S-gene candidates. This also implies that simple modification or disruption of such genes may negatively impact plant fitness. Therefore, it is critical to pinpoint precise editing sites that can disrupt effector recognition or binding without impairing the intrinsic function of the host protein. Achieving this goal requires in-depth mechanistic studies, especially structural analyses of effector-host protein interactions at the molecular level. Promisingly, recent advances in artificial intelligence (AI)-based protein structure prediction and complex modeling technologies are rapidly expanding our capacity to analyze these interactions with unprecedented accuracy, offering powerful tools to guide strategies to identify valuable genome-editing target sites for engineering durable resistance. A representative example is the Pc12 effector of *P. capsici* (Kim et al., 2024). A point mutation in the RAB13 protein that interrupt the interaction with Pc12 was guided by AlphaFold2-multimer (Mirdita et al., 2022) successfully abolished Pc12 interaction with Rab13 protein and attenuated *P. capsici* infection in *N. benthamiana* (Kim et al., 2024).

Research on effectors that specifically target intracellular organelles such as the chloroplasts, plasma membrane, mitochondria, ER, and Golgi apparatus in host cells remains relatively limited to date. Effector localization is still often inferred from sequence-based analyses, for example by identifying N-terminal signal sequences, lipid-based post-translational modifications, or transit peptides that are characteristics of proteins targeted to specific organelles (Sperschneider et al., 2017). Such *in silico* analyses, including chloroplast transit peptides and ER-retention motifs, provide useful initial insights into potential effector destinations, although experimental validation remains essential to confirm their subcellular localization and function.

As stated in the beginning of this review, several important effectors were not discussed in this review due to their cytosolic or nucleus localization or unclear localization and mode of actions. For examples, phytoplasma effectors, SAP11 and TENGU from Aster Yellows phytoplasma Witches’ Broom, predominantly localize to the nucleus or the cytosol and therefore were not discussed in this manuscript (Sugio et al., 2014; Omenge et al., 2021). Another effector candidate *Po*StoSP28 does not localize to the nucleus but targets the autophagosome. However, the effector localization was partially co-localized with the autophagosome markers which were not induced the autophagy, thereby cytosolic diffusion and some aggregates were shown (Dermastia et al., 2023).

Similarly, effectors from another fastidious phloem pathogen, *Liberibacter* spp., have been reported to predominantly target the cytosol, nucleus, or cytoplasmic membranes (Pitino et al., 2016; Zhang et al., 2023). Although several candidate effectors of ‘*Candidatus* Liberibacter asiaticus’ appear to form large aggregates in or near the chloroplast or nucleus, their molecular functions remain poorly characterized. For this reason, they were not addressed in this review. In the case of other fastidious phloem pathogens, such as *Spiroplasma*, we consider that current knowledge regarding effector targeting to host organelles is still limited and insufficient for meaningful discussion. Defining their organelle localization will be a good initiation to understand their mode of actions.

In conclusion, these subcellular compartments play indispensable roles in key processes such as energy metabolism, ROS production, protein homeostasis, lipid trafficking, and hormone biosynthesis, all of which are tightly linked to plant immunity. A deeper, integrated understanding of how effectors manipulate organelle functions will therefore be highly valuable. This research could enable the identification of novel resistance targets, the development of breeding strategies aiming to preserve organelle functions, and the design of advanced plant protection programs that reinforce organelle stability during pathogen attack. By positioning specific organelle-targeting effectors at the center of plant-pathogen research, future studies may uncover fundamental principles that can be translated into durable resistance in crops.
