# Correction: Pathogen effector tactics to suppress plant endomembrane system

**DOI:** 10.3389/fpls.2026.1944875

**Published:** 2026-07-31

**Authors:** Solhee In, Hye-Young Lee, Jongchan Woo, Doil Choi, Eunsook Park

**Affiliations:** 1 Department of Molecular Biology, College of Agricultural, Life Sciences and Natural Resources, University of Wyoming, Laramie, WY, United States; 2 Department of Agriculture, Forestry and Bioresources, Plant Genomics and Breeding Institute, College of Agriculture and Life Sciences, Seoul National University, Seoul, Republic of Korea; 3 Plant Immunity Research Center, College of Agriculture and Life Sciences, Seoul National University, Seoul, Republic of Korea; 4 Department of Horticulture, Gyeongsang National University, Jinju, Republic of Korea; 5 Institute of Agriculture & Life Science, Gyeongsang National University, Jinju, Republic of Korea; 6 Innocorelix, LLC., Laramie, WY, United States

**Keywords:** chloroplast, endoplasmic reticulum, organelle-mediated susceptibility, pathogen effector, plant endomembrane system

The funder “National Research Foundation of Korea (NRF) grants funded by the Korean government (MSIT)”, “2021R1C1C2006981” to “Hye-Young Lee” was erroneously omitted from the Funding statement.

In the **Acknowledgements** statement, **Funding** information “and 2021R1C1C2006981 to H-YL.” for H-YL is missing.

The original version of this article has been updated.

