# Supplementary figures and images for: Pathogen effector tactics to suppress plant endomembrane system
Source: Front Plant Sci. 2026 Mar 20;17:1776548. doi: 10.3389/fpls.2026.1776548 (PMC13047107; doi:10.3389/fpls.2026.1776548)

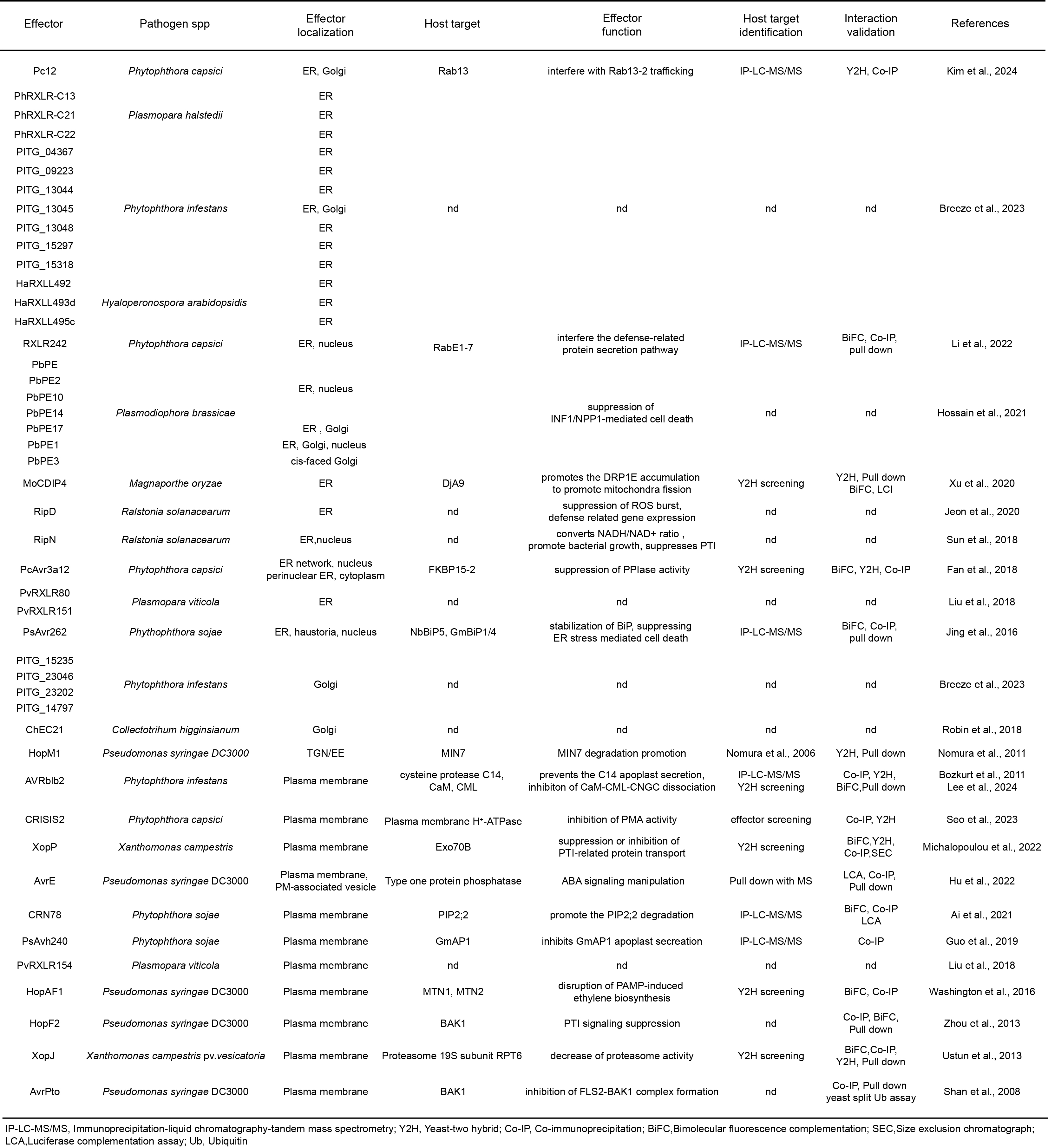

Supplement: Supplementary file 1 [file Image1.jpeg]

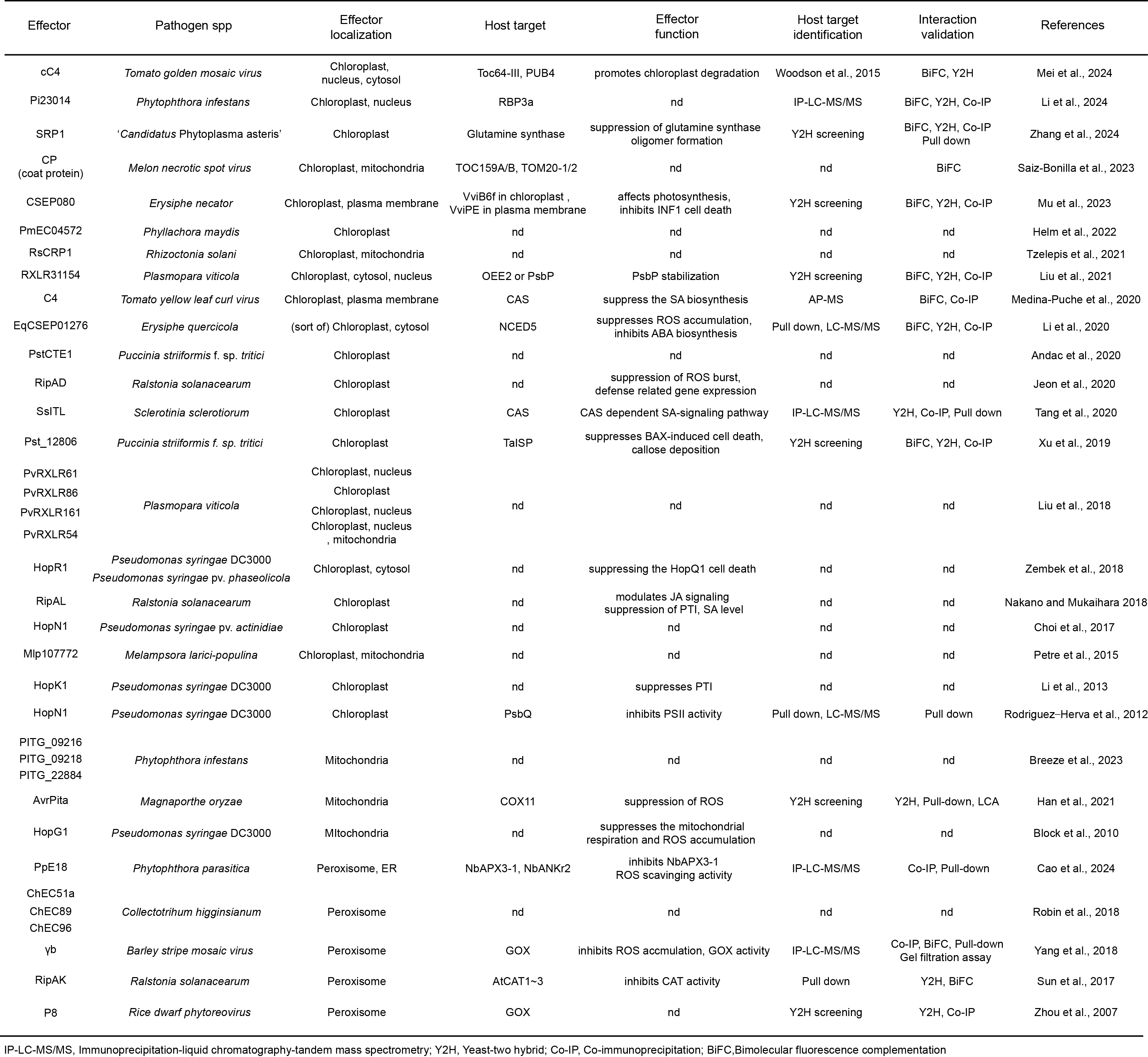

Supplement: Supplementary file 2 [file Image2.jpeg]
